# Supplementary material for: EnzML: multi-label prediction of enzyme classes using InterPro signatures
Source: BMC Bioinformatics. 2012 Apr 25;13:61. doi: 10.1186/1471-2105-13-61 (PMC3483700; doi:10.1186/1471-2105-13-61)
Supplement: Addtional file 5 — The Java code to format the data files, evaluate and predict. The file enzml_java_code.tar.gz contains the Java code used to format database data to ARFF and XML formats, to execute cross and train-test (jackknife) evaluations and to record evaluation results to database. More information is included in the readme.txt file and the Javadoc files. The code can be used with a MySQL database. To use a different database software, other JDBC drivers might be required. [file 1471-2105-13-61-S5.gz › java_code/enzml2011/doc/uk/ac/ed/inf/enzml/mulan/learn/class-use/MulanLearner.html]

Uses of Class uk.ac.ed.inf.enzml.mulan.learn.MulanLearner


---


|  |  |  |
| --- | --- | --- |
| **Overview** \| **Package** \| **Class** \| **Use** \| **Tree** \| **Deprecated** \| **Index** \| **Help** | |  |
| PREV   NEXT | **FRAMES**    **NO FRAMES**     **All Classes** |  |


---


## **Uses of Class uk.ac.ed.inf.enzml.mulan.learn.MulanLearner**

| Packages that use MulanLearner | |
| --- | --- |
| **test.mulan.learn** |  |
| **test.mulan.learn.traintest** |  |
| **uk.ac.ed.inf.enzml.mulan.learn** |  |
| **uk.ac.ed.inf.enzml.mulan.learn.traintest** |  |
| **uk.ac.ed.inf.enzml.mulan.predict** |  |

Uses of MulanLearner in test.mulan.learn

| Methods in test.mulan.learn that return MulanLearner | |
| --- | --- |
| `static MulanLearner` | `LearnerTest.getBRk1NN()` |
| `static MulanLearner` | `LearnerTest.getBRkNN(int neighbours)` |
| `static MulanLearner` | `LearnerTest.getMulanLearnerBRk1NN()` |
| `static MulanLearner` | `LearnerTest.getMulanLearnerOneR()` |
| `static MulanLearner` | `LearnerTest.getMulanLearnerZeroR()` |

Uses of MulanLearner in test.mulan.learn.traintest

| Methods in test.mulan.learn.traintest with parameters of type MulanLearner | |
| --- | --- |
| `static TrainTestExperimenter` | `TrainTestExperimenterTest.getMulanTrainTestExperById(int trainId, int testId, MulanLearner learner)` |

Uses of MulanLearner in uk.ac.ed.inf.enzml.mulan.learn

| Methods in uk.ac.ed.inf.enzml.mulan.learn that return MulanLearner | |
| --- | --- |
| `static MulanLearner` | `MulanLearner.getBRk1NN()` |
| `static MulanLearner` | `MulanLearner.getBRkNN(int neighbours)` |
| `MulanLearner` | `MulanCrossEvaluator.getLearner()` |
| `MulanLearner` | `MulanSerializer.getLearner()` |
| `MulanLearner` | `MulanCrossExperimenter.getLearner()`             public String getExperimentPropsPath() { return m\_experimentPropsFile; } |

| Methods in uk.ac.ed.inf.enzml.mulan.learn that return types with arguments of type MulanLearner | |
| --- | --- |
| `static java.util.Vector<MulanLearner>` | `MulanLearners.getLearners()` |

| Methods in uk.ac.ed.inf.enzml.mulan.learn with parameters of type MulanLearner | |
| --- | --- |
| `static uk.ac.ed.inf.utils.database.TableRow` | `ResultsFormatter.createEvaluationRecord(int runNumber, int trainArffId, int testArffId, java.lang.String startTime, java.lang.String endTime, MulanLearner learner, mulan.evaluation.Evaluation evaluationResults, uk.ac.ed.inf.utils.database.TableManager tableManager)`             Add timestamps and identifiers to the cross evaluation results |
| `static java.lang.String` | `MulanSerializer.run(int trainArffId, MulanLearner learner, java.lang.String arffDatabaseProps, java.lang.String folderForModel)` |
| `int` | `ResultsSaver.saveResults(int runNumber, int trainArffId, int testArffId, java.lang.String startTime, java.lang.String endTime, MulanLearner learner, mulan.evaluation.Evaluation evaluationResults)` |

Constructors in uk.ac.ed.inf.enzml.mulan.learn with parameters of type MulanLearner

`MulanCrossExperimenter(java.lang.String resultsDbPropsFile,
java.lang.String arffRecordDbProps,
int trainArffRecordId,
MulanLearner learner,
java.lang.String resultsFolder)`
  
          public MulanCrossExperimenter(String resultsDbPropsFile, String
arffRecordDbProps, MulanArffRecord trainArffRecord, MulanLearner learner,
String resultsFolder) { m\_trainArffRecord = trainArffRecord;
this.initialise(resultsDbPropsFile, arffRecordDbProps, learner,
resultsFolder); }

`MulanSerializer(MulanArffRecord arffRecord,
MulanLearner learner,
java.lang.String directoryForSerializedModel)`

Uses of MulanLearner in uk.ac.ed.inf.enzml.mulan.learn.traintest

| Methods in uk.ac.ed.inf.enzml.mulan.learn.traintest with parameters of type MulanLearner | |
| --- | --- |
| `static void` | `TrainTestExperimenterSerialized.run(java.lang.String resultsDbProps, java.lang.String arffRecordDb, int trainArffRecordId, int testArffRecordId, MulanLearner learner, java.lang.String trainedModelFile, java.lang.String resultsFilesDirectory)` |

Constructors in uk.ac.ed.inf.enzml.mulan.learn.traintest with parameters of type MulanLearner

`TrainTestExperimenter(java.lang.String experimentPropsFile,
java.lang.String arffRecordDbProps,
int trainArffRecordId,
int testArffRecordId,
MulanLearner learner,
java.lang.String resultsDirectory)`

`TrainTestExperimenterSerialized(java.lang.String experimentPropsFile,
java.lang.String arffRecordDb,
int trainArffRecordId,
int testArffRecordId,
MulanLearner learner,
java.lang.String trainedModelFile,
java.lang.String resultsDirectory)`

Uses of MulanLearner in uk.ac.ed.inf.enzml.mulan.predict

| Methods in uk.ac.ed.inf.enzml.mulan.predict with parameters of type MulanLearner | |
| --- | --- |
| `static java.lang.String` | `MulanPredict.run(int trainId, int testId, java.lang.String machineLearningProps, java.lang.String predictionsDirectory, MulanLearner learner)`             Generates and saves predictions. |

Constructors in uk.ac.ed.inf.enzml.mulan.predict with parameters of type MulanLearner

`MulanPredict(int trainArffId,
int unlabeledArffId,
java.lang.String arffRecordsDatabase,
java.lang.String predictionsDirectory,
MulanLearner learner)`

`MulanPredictWithTrainedModel(int trainArffId,
int unlabeledArffId,
java.lang.String arffRecordsDatabase,
java.lang.String predictionsDir,
java.lang.String builtTrainedModel,
MulanLearner learner)`

---


|  |  |  |
| --- | --- | --- |
| **Overview** \| **Package** \| **Class** \| **Use** \| **Tree** \| **Deprecated** \| **Index** \| **Help** | |  |
| PREV   NEXT | **FRAMES**    **NO FRAMES**     **All Classes** |  |


---
